# Brain-type and liver-type fatty acid-binding proteins: new tumor markers for renal cancer?

**DOI:** 10.1186/1471-2407-9-248

**Published:** 2009-07-21

**Authors:** Angelika Tölle, Monika Jung, Michael Lein, Manfred Johannsen, Kurt Miller, Holger Moch, Klaus Jung, Glen Kristiansen

**Affiliations:** 1 Department of Urology, Charité – Universitätsmedizin Berlin, Berlin, Germany; 2 Berlin Institute for Urologic Research, Berlin, Germany; 3 Institute of Surgical Pathology, Universitätsspital Zürich, Zurich, Switzerland

## Abstract

**Background:**

Renal cell carcinoma (RCC) is the most common renal neoplasm. Cancer tissue is often characterized by altered energy regulation. Fatty acid-binding proteins (FABP) are involved in the intracellular transport of fatty acids (FA). We examined the level of brain-type (B) and liver-type (L) FABP mRNA and the protein expression profiles of both FABPs in renal cell carcinoma.

**Methods:**

Paired tissue samples of cancerous and noncancerous kidney parts were investigated. Quantitative RT-PCR, immunohistochemistry and western blotting were used to determine B- and L-FABP in tumor and normal tissues. The tissue microarray (TMA) contained 272 clinico-pathologically characterized renal cell carcinomas of the clear cell, papillary and chromophobe subtype. SPSS 17.0 was used to apply crosstables (χ^2^-test), correlations and survival analyses.

**Results:**

B-FABP mRNA was significantly up-regulated in renal cell carcinoma. In normal tissue B-FABP mRNA was very low or often not detectable. RCC with a high tumor grading (G3 + G4) showed significantly lower B-FABP mRNA compared with those with a low grading (G1 + G2). Western blotting analysis detected B-FABP in 78% of the cases with a very strong band but in the corresponding normal tissue it was weak or not detectable. L-FABP showed an inverse relationship for mRNA quantification and western blotting. A strong B-FABP staining was present in 52% of the tumor tissues contained in the TMA. In normal renal tissue, L-FABP showed a moderate to strong immunoreactivity in proximal tubuli. L-FABP was expressed at lower rates compared with the normal tissues in 30.5% of all tumors. There was no correlation between patient survival times and the staining intensity of both FABPs.

**Conclusion:**

While B-FABP is over expressed in renal cell carcinoma in comparison to normal renal tissues L-FABP appears to be reduced in tumor tissue. Although the expression behavior was not related to the survival outcome of the RCC patients, it can be assumed that these changes indicate fundamental alterations in the fatty metabolism in the RCC carcinogenesis. Further studies should identify the role of both FABPs in carcinogenesis, progression and with regard to a potential target in RCC.

## Background

In adults, renal cell carcinoma (RCC) represents about 4% of all malignant solid tumors. In 2008, RCC was expected to result in 54390 new cases and in 13010 deaths in the USA [1]. The prognosis of patients with distant metastasis is very poor with a 5-year survival rate of less than 10% [2], whereas patients with tumor stages pT1 and pT2 show a survival rate of 80–90% during the first 5 years after diagnosis [3]. An early diagnosis renders curative surgery possible and thus improves prognosis. Therefore, novel biomarkers are needed, firstly, as tools to detect tumors early and secondly, as therapeutic targets to improve treatment options [4,5].

Cancer is characterized by an altered energy regulation. Fatty acid-binding proteins (FABPs) are involved in the uptake, the intracellular transport, and the delivery of fatty acids to beta-oxidation. FABPs are also important in cell signaling, regulation of gene expression, cell growth, and differentiation [6]. Currently, nine members of the FABP family have been identified named after the first tissue of isolation: (a) liver (L-FABP); (b) intestinal (I-FABP); (c) heart (H-FABP); (d) adipocyte (A-FABP); (e) epidermal (E-FABP); (f) ileal (IL-FABP); (g) brain (B-FABP); (h) myelin (M-FABP) and (i) testis (T-FABP) [7].

The importance of FABPs for the progression of carcinomas was shown for prostate cancer [8], breast cancer [9], bladder cancer [10] and astrocytomas [11]. Search in free available mRNA data bases revealed the brain-type FABP (B-FABP) as strongly over-expressed in RCC [12]. A heterogeneous expression pattern of various members of the FABP-family was demonstrated in RCC by immunoblotting and RT-PCR analyses [13]. In healthy and benign kidney parenchyma, L-FABP is localized in proximal tubules [14,15].

However, these data obtained from a limited number of cases did not allow a clear conclusion with regard to the clinical usefulness of these potential markers. Therefore, the objectives of the present study were related to clear cell RCC (ccRCC), the most common (75% of cases) renal cancer subtype with the most serious prognosis [16]. The study was aimed (a) to compare expression of B-FABP and L-FABP on protein and transcript level in noncancerous areas and RCC lesions of surgically resected kidneys, (b) to correlate these expression data with clinico-pathological parameters concerning its diagnostic value, and (c) to evaluate the immunohistochemical staining data of B-FABP on a RCC tissue-microarray with the survival outcome of RCC patients.

## Methods

### Patients (reverse transcriptase-polymerase chain reaction)

Forty-eight matched (malignant and nonmalignant) specimens from kidney were used for total RNA isolation. The samples were derived from patients with RCC undergoing radical nephrectomy at the Department of Urology, Charité – Universitätsmedizin Berlin, Germany. Staging met the UICC 2002 criteria. Histological classification was performed according to the WHO criteria, tumor grading was accomplished according to Fuhrman. The Fuhrman grades were G1 in 3 (6.2%), G2 in 31 (64.6%), G3 in 12 (25%) and G4 in 2 (4.2%) cases respectively. This cohort enclosed patients without (n = 28) and with metastasis (n = 20). Tissue materials used for RNA isolation differed from the cohort used for immunohistochemistry. This study has been approved by the Charité University Ethics Committee.

### Patients (immunohistochemistry)

A tissue microarray (TMA) was constructed from renal cell carcinomas diagnosed at the Institute of Surgical Pathology, Universitätsspital Zurich, between 1993 and 2003. The Ethics Committee of Universitätsspital Zurich has been approved the study. Cases were selected according to tissue availability, without any further stratification for clinical or pathological prognostic factors. Two-hundred seventy two renal carcinomas were represented on the TMA. Staging and grading criteria corresponded to the criteria described above. The median patient age of these patients was 63.7 years (range: 29 to 88 years). 177 patients were men, 95 women.

The majority of the carcinomas (n = 224, 82.4%) were of clear cell (cc) type. The remaining RCC were of papillary (n = 37; 13.6%), and chromophobe (n = 11; 4.0%) types. The pT-status for these cases was as follows: pT1 - 112 (41.2%), pT2 - 35 (12.9%), pT3 - 120 (44.1%), pT4 - 5(1.8%).

The Fuhrman grades were G1 in 4 (1.5%), G2 in 87 (32.0%), G3 in 114 (41.9%) and G4 in 67 (24.6%) cases respectively. Disease specific death occurred in 115 patients (42.3%) after a median survival time of 48.8 months (range 0–140) (Table 1). In the TMA, each tumor was represented by one tissue core (0.6 mm). Data on the metastatic state were available from 139 patients only.

**Table 1:** Clinico-pathological parameters (percentages in brackets) and protein expression of B- and L-FABP in renal cell carcinomas.

		Total	B-FABP positive	L-FABP positive
All cases		272 (100)	141 (51.8)	83 (30.5)
Age		29 – 88		
mean		63.7		
Histology	Clear cell	224 (82.4)	123 (54.9)	69 (30.8)
	Papillary	37 (13.6)	11 (29.7)	10 (27.0)
	Chromophobe	11 (4.0)	7 (63.4)	4 (36.4)
				
Fuhrman Grading	G1	4 (1.5)	3 (75.0)	1 (25.0)
	G2	87 (31.9)	44 (50.6)	29 (33.3)
	G3	114 (42.0)	63 (55.3)	34 (29.8)
	G4	67 (24.6)	31 (46.3)	19 (28.3)
				
pT status	pT1	112 (41.2)	58 (51.8)	38 (33.9)
	pT2	35 (12.9)	16 (45.7)	7 (20.0)
	pT3	120 (44.1)	62 (51.7)	37 (30.8)
	pT4	5 (1.8)	5 (100)	1 (20.0)
				
Metastasis	M0	96 (35.3)	49 (51.0)	32 (33.3)
	M1	42 (15.4)	24 (57.1)	9 (21.4)
	X	134 (49.3)	68 (50.7)	42 (31.3)

### Quantitation of B-FABP and L-FABP mRNA

For RT-PCR, matched malignant and non-malignant specimens from the same kidney were collected immediately after surgery in RNAlater^® ^Stabilization Reagent (Qiagen, Hilden, Germany), stored overnight at 4°C and at -80°C afterwards. Total RNA was isolated from about 40 mg tissue samples using the RNeasy Mini Kit (Qiagen) according to the manufacturer's instructions including an additional genomic DNA digestion step with DNase I (Qiagen). The RNA yield and quality were determined using the NanoDrop^® ^ND-1000 Spectrophotometer (NanoDrop Technologies, Montchanin, USA) and the Agilent 2100 Bioanalyzer with RNA 6000 Nano LabChips^® ^(Agilent Technologies, Palo Alto, USA).

One μg RNA was reverse transcribed using the Transcriptor First Strand cDNA Synthesis Kit (Roche Applied Science, Mannheim, Germany) by random hexamer priming method according to the manufacturer's recommendations. RNA samples were controlled for genomic contamination by omitting the reverse transcriptase in the cDNA synthesis mixture. Real-time RT-PCR was performed with the LigthCycler^® ^480 Instrument (Roche) equipped with a 96-well block. The B-FABP (Accession number: NM_001446) and L-FABP (Accession number: NM_001443) specific PCR assay was designed with the web-based ProbeFinder software Version 2.40 for Human http://www.universalprobelibrary.com using primers and probe of the Universal ProbeLibrary (UPL; Roche). We selected for the B-FABP real-time PCR the UPL assay with folllowing primer-probe combination: the forward primer 5'-ctcagcacattcaagaacacg-3' and the reverse primer 5'-ccatccaggctaacaacagac-3' with the UPL probe #33 (Roche cat. no. 04687663001). For the L-FABP real time PCR the UPL assay with the following primer-probe combination was selected: the forward primer 5'-ttctccggcaagtaccaact-3' and the reverse primer 5'-cttccccttctggatgagc-3' with the UPL probe #72 (Roche cat. no 04688953001).

A 10 μl PCR reaction mix included 0.25 μmol/l of each primer and 0.1 μmol/l probe as final concentration, 1 μl undiluted cDNA and the ready-to-use LigthCycler 480 Probes Master Probe (Roche). The PCR run conditions were: activation of Taq DNA polymerase at 95°C for 10 min and 45 amplification cycles at 95°C for 10 s, 60°C for 15 s and 72°C for 1 s. The amplificon size was 104 bp. Each plate for PCR run included the cDNA samples from malignant and non-malignant tissue pairs, a non-template control, samples of a cDNA pool used as calibrator, and another one for precision control. For relative quantification of B-FABP mRNA expression, we also determined the two genes TATA box-binding protein (TBP) and peptidylproline isomerase A (PPIA) verified in a previous study as most suitable reference genes for gene profiling studies in renal cell carcinoma [17]. For all three gene-specific PCRs, standard curves were generated with pooled cDNA samples for calculation of gene expression and PCR efficiencies. All samples were measured in duplicates.

### Western blot analysis for B-FABP and L-FABP

Protein extraction and western blotting technique were performed as described previously [18]. Paired tissue samples (renal cancer/normal) from fourteen nephrectomy specimens were used. Western blotting was carried out with the rabbit polyclonal anti-human B-FABP antibody and rabbit polyclonal anti-human L-FABP antibody (1 μg/ml; Hycult biotechnology b.v., Uden, Netherlands). Horseradish peroxidase-conjugated goat anti-rabbit IgG (DakoCytomation, Glostrup, Denmark) was used as secondary antibody. Actin (anti-β-actin clone AC-74, Sigma-Aldrich Chemie GmbH, Munich, Germany) served as loading control and secondary antibody was horseradish peroxidase-conjugated rabbit anti-mouse IgG (DakoCytomation). The antigen-antibody reaction was visualized by ECL Advance™ Western Blotting Detection Kit (GE Healthcare UK Limited, Little Chalfont Buckinghamshire, UK). Intensity of the detected signals by Western blot was quantified with Fluor-S MultiImager (Bio-Rad Laboratories, Hercules, USA).

### Tissue microarray immunohistochemistry

The TMA blocks were freshly cut (3 μm) and mounted on superfrost slides (Menzel Gläser, Braunschweig, Germany). Immunohistochemistry was conducted with the Ventana Benchmark automated staining system (Ventana Medical Systems, Tucson, AZ, USA) using Ventana reagents for the entire procedure. The primary antibodies were rabbit polyclonal anti-human B-FABP antibody and rabbit polyclonal anti-human L-FABP antibody (Hycult) were used at a concentration of 1 μg/ml. For detection we used the UltraVIEW™ DAB detection kit using the benchmarks CC1m- heat induced epitope retrieval. Slides were counterstained with hematoxylin, dehydrated and mounted.

### Evaluation of immunohistochemical stainings

**Figure 1:**
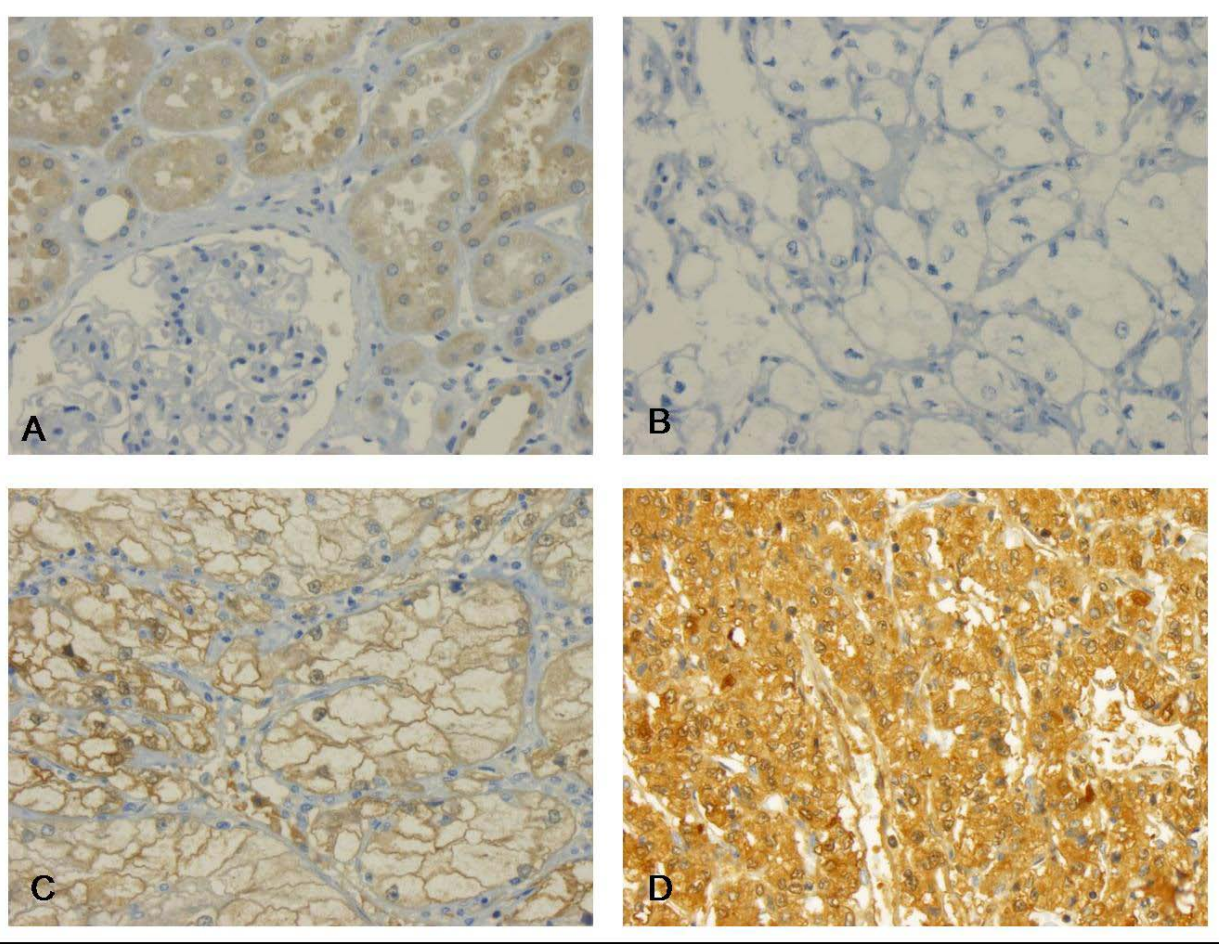
**B-FABP immunohistochemistry**. **(A) **In normal tissue B-FABP is preferentially expressed in proximal tubuli. **B-D **Clear cell carcinomas without **(B)**, weak to moderate immunoreactivity **(C) **and strong expression of B-FABP **(D)**.

**Figure 2:**
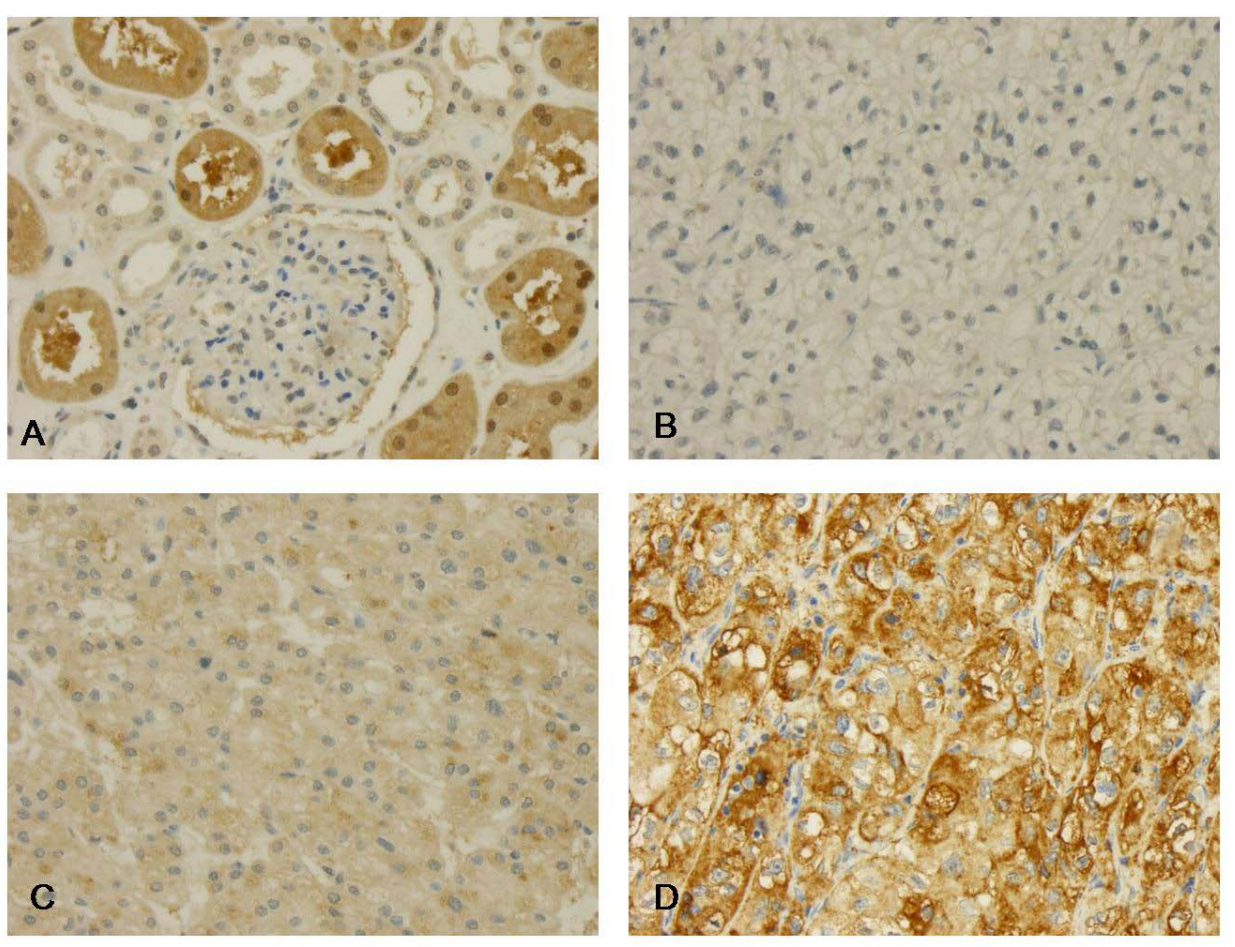
**L-FABP immunohistochemistry**. **(A) **In normal tissue L-FABP is preferentially expressed in proximal tubuli. **B-D **Clear cell carcinomas without **(B)**, weak to moderate immunoreactivity **(C) **and strong expression of L-FABP **(D)**.

The immunohistochemistry was evaluated by a single pathologist (GK) in one go to minimize intraobserver variability. Intensity of immunoreactivity was semiquantitatively scored as negative, weakly, moderately or strongly positive as illustrated by Figure 1 and 2. This panel of figures was compiled before the systematic evaluation of the tumor cohort was commenced and was used as reference, which lay next to the microscope for continuous comparison.

### Statistical analysis

Statistical analysis was performed with SPSS, version 17.0 (SPSS Inc, Chicago, IL, USA). P values < 0.05 were considered significant. Sample size determinations and power calculations were performed using the software's GraphPad Statmate for Windows, version 2.0 (GraphPad Software) and MedCalc, version 10.0.2 (Mariakerke, Belgium) on the basis of a two-sided alpha error of 5% and a power of 80%. Assuming a proportional difference of about 0.45 in Kaplan-Meier curves as observed in RCC patients due to the main risk factor tumor stage [19], about 25 subjects in each group have to be investigated to show corresponding prognostic information of FABP expression. Instead of using 25 subjects in each group, the ratio of subjects between the two groups could be increased to 3 without losing statistical power, if the total number of patients is increased to 68. Thus, since the sample size between the groups could not be predicted, at least this number of patients should be included in the follow-up study to guarantee a power not less than 80% as mentioned above.

## Results

### Quantitative reverse transcriptase-PCR

The RNA purity was given by an absorbance 260 nm/280 nm ratio in range over all RNA samples from 1.98 to 2.10. The RNA integrity was characterized by the RIN value of 8.4 ± 1.03 (mean ± SD). The precision of the PCR measurements amounted to a variation coefficient of 0.46% at a mean Cp-value of 26.8.

**Figure 3:**
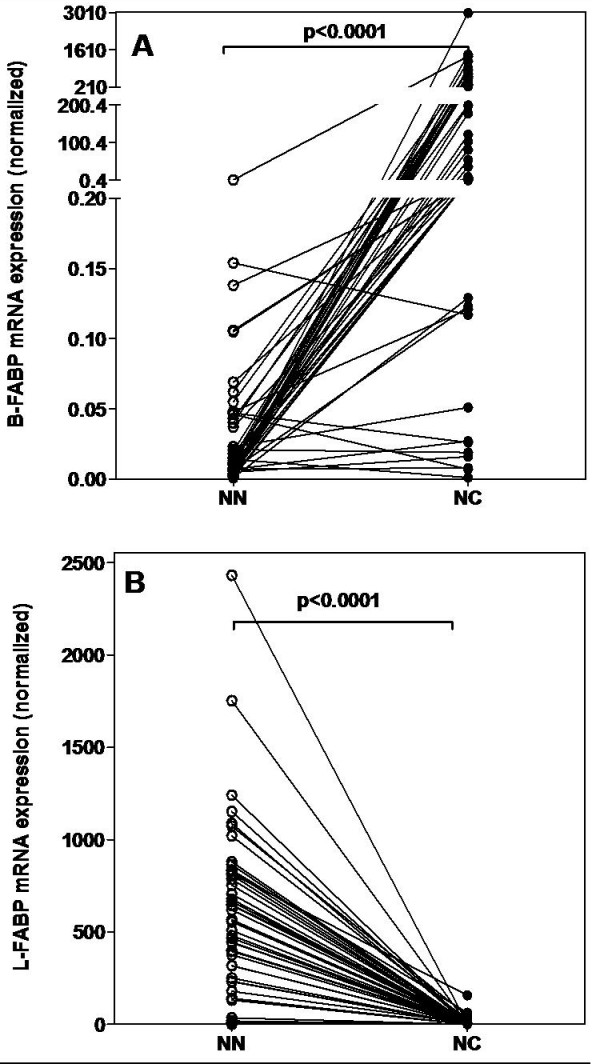
**FABP mRNA expression**. FABP mRNA expression in matched normal (NN) and malignant (NC) RCC tissue samples. Expression data were normalized against the geometric means of the two reference genes PPIA and TBP. Statistical differences were calculated by the Wilcoxon test. **(A) **B-FABP, **(B) **L-FABP mRNA expression.

The expressions of B- and L-FABP in normal and tumor tissues were normalized against the geometric mean expression of the two reference genes PPIA and TBP. The relative B-FABP mRNA expression was 2759-fold higher (median) in RCC than in the normal tissue (p < 0.0001; Figure 3A). The relative B-FABP mRNA expression depended on the tumor grade. Samples of the grades G1 and G2 (34 cases) displayed a 6410-fold (median) over-expression, whereas the samples of the grades G3 and G4 showed only a 39-fold (median) over-expression.

L-FABP showed inverse results. The relative L-FABP mRNA expression was high in the normal kidney tissues, but very low in RCC samples. The relative L-FABP mRNA expression was 179-fold lower (median) in RCC than in normal tissue (p < 0.0001; Figure 3B). The down regulation of L-FABP in tumors with a high grading (G3 and G4) was significantly higher (p = 0.035) than in tumors with a low grading (G1 and G2). Samples of the grades G1 and G2 displayed a 120-fold (median) down regulation and samples of G3 and G4 showed a 300-fold (median) decrease.

### Western blot

**Figure 4:**
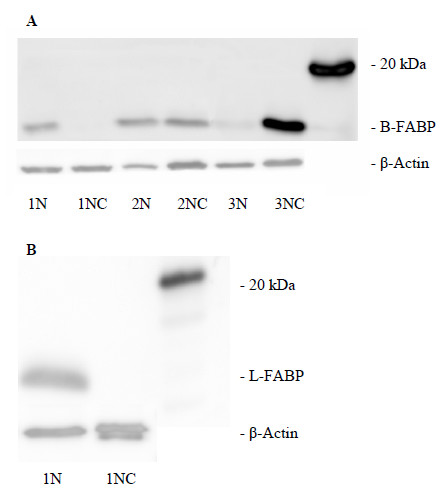
**Western blot detection of FABP**. FABP and β-Actin in matched normal (NN) and malignant (NC) RCC tissue samples. **(A) **B-FABP, **(B) **L-FABP.

The B- and L-FABP protein expression was standardized against β-Actin's expression. Then a ratio of tumor to normal tissue was calculated. The B-FABP expression in tumor tissues was classified in three groups. For six patients the calculated ratio was higher 2. The ratio was higher 1 and lower 2 for five patients and only three patients showed a ratio lower 1. Figure 4 illustrates representative blot of the three B-FABP groups and of L-FABP protein expression in normal and renal tumor extracts. Out of the 14 patients investigated, 11 patients (78%) expressed B-FABP in tumor tissue. In the corresponding normal tissues, the B-FABP was negligible or very weak. An association between the B-FABP protein expression and the tumor stage or grade was not calculated because of the small number of samples (Figure 4A).

The L-FABP was detectable in all (14) normal renal tissue lysates, but in none RCC sample (Figure 4B) so that only ratios lower 1 were calculated.

### FABP immunostaining in normal and malignant renal tissues

In normal renal tissue, B-FABP showed a weak immunoreactivity in proximal tubuli (Figure 1A). Few distal tubuli were also inconsistently positive. No immunoreactivity was observed in glomeruli (Figure. 1B). In 52% of all tumors, B-FABP was expressed at higher rates, showing a predominantly cytoplasmic, often membrane accentuated staining pattern (Figure 1C–D). Nuclei were also positive in some cases, but this was not subjected to systematic evaluation. The immunoreactivity was present in all tumor subtypes.

In normal renal tissue, L-FABP showed a moderate to strong immunoreactivity in proximal tubuli (Figure 2A). Distal tubuli stained weakly (Figure 2C), no immunoreactivity was observed in glomeruli (Figure 2B). In 30.5% of all tumors, L-FABP was expressed at lower rates, showing a purely cytoplasmic staining pattern.

### Association between clinico-pathological parameters and FABP immunostaining data

As expected, tumor stage and grade were strongly correlated (r_s _= 0.372; p < 0.0001), however, no associations were found between both parameters and protein expression of B-FABP and L-FABP (r_s _= -0,041 – 0.07; p = 0.25 – 0.655).

### FABP expression and patient survival time

**Figure 5:**
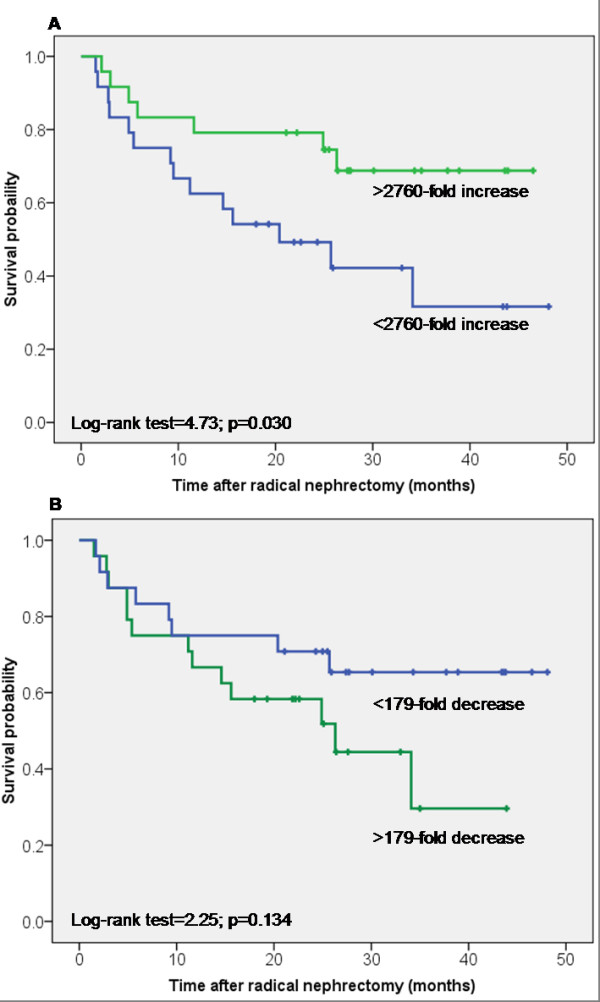
**Kaplan-Meier survival curves for B- and L-FABP mRNA expression**. The mRNA over-expression above and below the median was compared for all cases. **(A) **for B-FABP and **(B) **for L-FABP.

First, Kaplan-Meier analyses were performed with the mRNA expression data

(Figure 5). The results of the survival times correspond to the above mentioned relationships between both FABP expressions and the tumor grade as well as and the well-known fact that patients with high-grade tumors have a lower survival times. That effect is already statistically significant (for L-FABP Log-rank test, p = 0.038) or evident in tendency (for B-FABP Log-rank test, p = 0.107) in patients with tumor grade G1 and G2 whereas there were no correlations in those with the high tumor grades G3 and G4 (Log-rank test, p = 0.241 and p = 0.919, respectively). Using the multivariable Cox regression analysis with the variables pT stage, grade, and FABPs, the two FABPs did not arise as independent variables (Log-rank test, p = 0.283 for B-FABP; and Log-rank test, p = 0.945 for L-FABP). In a final selective model after a forward elimination procedure, only tumor grade remained as significant parameter (hazard ratio of 5.59, p < 0.0001).

**Figure 6:**
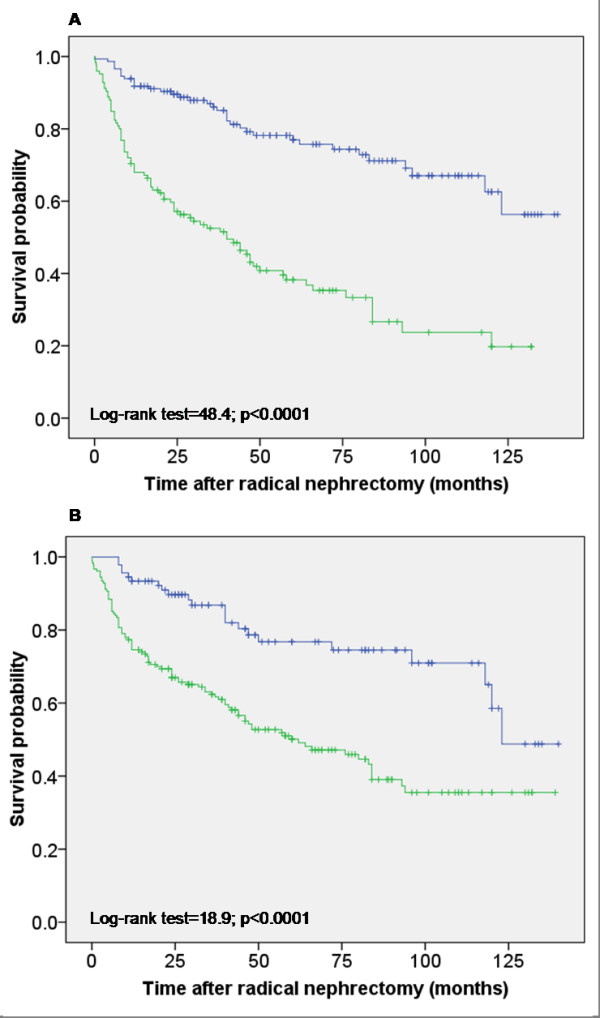
**Kaplan-Meier survival curves for tumor stage and Fuhrman grades in the TMA cohort**. **(A) **Survival curves for tumor stage. Tumors with stage 1 and 2 (blue line) revealed significantly longer survival times if compared to those with high stage 3 and 4 (green line). **(B) **Survival curves for Fuhrman grades. Tumors with grade 1 and 2 (blue line) revealed significantly longer survival times if compared to those with high grade 3 and 4 (green line).

**Figure 7:**
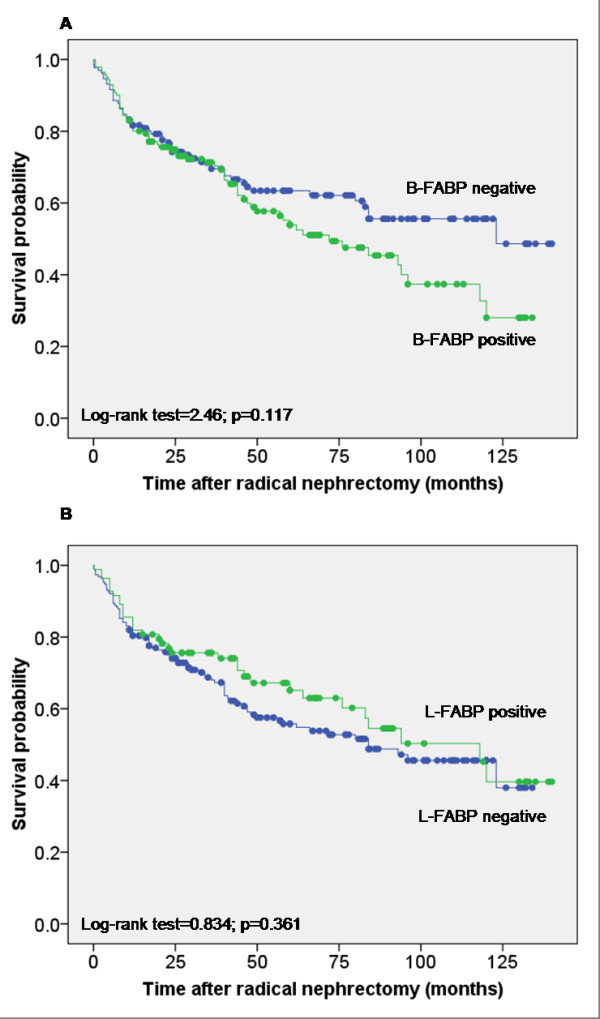
**Kaplan-Meier survival curves for B- and L-FABP expression in the TMA cohort**. Positive and negative FABP expression was compared for all cases. **(A) **B-FABP expression and **(B) **L-FABP expression.

In the following, we tested the prognostic significance of both FABP expressions by immunohistochemical staining using, from the practical point of view, the convenient TMA. The conventional prognostic parameters (Fuhrman grades, pT status, metastasis) reached significance for survival in Kaplan-Meier analysis (Log-rank test; p < 0.0001 for all these parameters) showing that our tumor cohort was representative (Figure 6). More than fifty percent of the RCC cases showed a B-FABP staining of different intensity. This staining (all intensities together) was not significantly associated with a shorter survival time (Log-rank test, p = 0.117) (Figure 7A). The same was seen in the subgroup of ccRCC (Log-rank test, p = 0.236), in papillary RCC (Log-rank test, p = 0.715) and in chromophobe RCC (Log-rank test, p = 0.718).

Similarly, L-FABP immunostaining was not significant related to survival time (Log-rank test, p = 0.361) for the total group (Figure 7B) and for the subgroups. If the group of ccRCC patients was stratified with regard to grading G1 and G2 respectively stage 1+2 and 3+4 there would not be significantly difference in survival time depended on B-FABP detection (Log-rank test, p = 0.231 respectively p = 0.278). Under the same conditions of stratification the L-FABP detection did also not influence significantly the survival time of patients (Log-rank test, p = 0.575 respectively p = 0.247).

We also tested the association of FABP expression and survival rate in the patient groups with and without metastasis. Reliable information on metastasis was only available from 139 patients and 96 patients were without metastasis. This group contained 79 clear cell, 13 papillary and 4 chromophobe type RCCs (Table 1). Because of the low number of the latter two RCC types only Kaplan-Meier analysis was done for ccRCC. In this subgroup of patients, staining of B- and L-FABP was not related to a shorter survival time (Log-rank-test, p = 0.27 and p = 0.96, respectively). Also in the subgroup of ccRCC patients with distant metastases (M1; n = 42) B- and L-FABP were not significantly associated with a shorter survival time (Log-rank test, p = 0.97 and p = 0.74, respectively).

## Discussion

The kidney is one of the organs that most actively metabolizes hydrophobic ligands. Also, the markedly increased total lipid content in renal cell carcinomas responsible for their grossly yellow appearance is considered a diagnostic hallmark of this tumor type [20]. Therefore, it did not come as a surprise when recently up-regulation of fatty acid synthase (FAS) in RCC with advanced pathological T stages was demonstrated [21]. This finding further underscores the high metabolic activities of RCC. B- and L-FABP are cytosolic proteins of the fatty acid binding protein family as briefly mentioned in the introduction. The FABP immunostaining in our TMA is a predominantly cytoplasmic, often membrane accentuated staining pattern. Nuclei are also positive in some cases, but were not taken into further consideration. At the inner side of the cell membrane, fatty acids are bound to cytoplasmic FABPs. They may actively facilitate the transport of lipids to specific compartments in the cell.

Movement of FABPs into the nucleus and interaction with nuclear hormone receptors (i.e. PPAR) is possible [7,22]. In normal renal tissue, L-FABP showed a moderate to strong immunoreactivity in proximal tubuli [14]. These proteins are responsible for the uptake and transport of bioactive lipids within the cells. The FABPs can bind many different groups of fatty acids and their derivates. FABPs were extensively investigated for various diseases such as acute and chronic myocardial injury [23,24], lung injury [25,26], psoriasis [27] and chronic kidney disease [28,29]. Changes of the different FABP subtypes were further shown for various tumors like prostate [8], breast [9], and bladder cancer [10] as well as astrocytomas [11].

We analyzed the B-FABP expression on the protein and transcript level in noncancerous areas and RCC lesions of surgically resected kidneys. B-FABP was over-expressed in mRNA-level in renal cell carcinoma in comparison to normal renal tissues (p < 0.0001). In tumors with a low grading (G1 and G2), the B-FABP mRNA was particularly high, whereas expression levels of B-FABP-mRNA were lower in poorly (G3 and G4) differentiated tumors. On protein level, B-FABP is detectable in 78% of cases in RCC tissues. Conversely, the L-FABP-mRNA was highly expressed in normal renal tissue and not detectable in RCC tissues independent on tumor grading and staging. These mRNA data corresponded with the western blot analyses as L-FABP was exclusively detectable in all (14) normal renal tissue lysates but not in RCC samples. These results provide additional evidence of altered fatty acid metabolism in RCC since the preferred FABP-type localized in the proximal tubules of the normal kidney is substituted in renal carcinomas [14]. Changed expression of FABPs was also found in other organs and cell lines. Hammamieh [8,9] observed an increase of L-and I-FABP in prostate and breast cancer cell lines, while A- and E-FABP were reduced in these cells compared to normal cells. Experimental blocking L-FABP resulted in the activation of antiproliferative genes like TNF-α and the induction of apoptosis. However, in contrast to the reduced E-FABP expression data in the cell lines, an over-expression of E-FABP was found in prostate carcinoma tissue [30]. More differentiated bladder squamous cell carcinomas exhibited higher level of E-FABP than less-differentiated tumors [31].

Teratani et al. [32] suggested the use of B-FABP transcripts as a novel urine marker for early detection of RCC and for monitoring RCC patients postoperatively. They also showed a significant over-expression of B-FABP gene in RCC samples depending on the tumor stage. However, our results based on matched pairs of malignant and non-malignant samples from the same tumor proved that the tumor grade more distinctly influence the expression. So it was possible to demonstrate that samples of the grades G1 and G2 displayed a 6410-fold (median) over-expression, whereas samples of the G3 and G4 showed only a 39-fold (median) over-expression. By immunoblotting and RT-PCR analyses, a heterogeneous expression pattern of various members of the FABP-family was demonstrated in RCC [13]. However, all these data obtained from a limited number of RCC cases did not allow a clear conclusion with regard to the clinical usefulness of these potential markers and an extensive analysis was pending.

In our study we utilized a tissue micro-array with 272 fully characterized cancer cases to provide reliable data on the immunohistochemical staining pattern of both FABPs in relation to clinico-pathological parameters and on the prognostic potential of B- and L-FABP. Our Kaplan-Meier and Cox regression analyses with the limited number of mRNA expressions data showed that both FABPs were no independent prognostic variables for the survival of patients. The immunohistochemical analyses also demonstrated that about fifty-five percent (54.9%) of the ccRCC cases showed a B-FABP staining of different intensity but not advantage was detected concerning survival times. While the conventional prognostic markers tumor stage and tumor grade were confirmed as risk factors in our study group, the over-expression of B-FABP was not an independent prognostic marker. These results partly contrast with the data of other FABP types from other cancer types. For example, Ohlsson [10] was able to demonstrate with a TMA containing more then 2,000 cases that the loss of A-FABP in urothelial carcinomas was associated with tumor progression. These results generally suggest that FABPs could have prognostic potential at least in combination with other biomarkers.

Above all, the metabolism of fatty acids respectively lipids is markedly changed in tumors, confirming the early observation of Warburg that tumor cells are characterized by an altered energy metabolism. The over-expression suggests that B-FABP might be a potential therapeutic target in RCC. Recently, small series of A-FABP inhibitors have been identified [7]. One member of the FABP family, L-FABP is controlled by peroxisome proliferator-activated receptor (PPAR) family. L-FABP interacts with PPARα and PPARγ by protein-protein contacts [33] and affects, in addition to the fatty acid metabolism, drug signaling regarding the PPARα activation. That effect could be assumed as general principle of the interaction of the different FABP types with the PPAR isoforms. Since expression of PPAR isoforms are commonly dysregulated in human carcinomas [34] and novel therapeutic options are provided by the anti-diabetic drug class of thiazolidindiones, further studies are clearly warranted to identify the role of B- and L-FABP in relation to PPAR isoforms in carcinogenesis and progression of renal cancer.

## Conclusion

Our results showed a differential expression of B-FABP and L-FABP in RCC samples both on the transcriptional and protein level. B-FABP was highly over-expressed in RCC whereas L-FABP was significantly reduced. Although the expression behavior was not related to the survival outcome of the RCC patients, it can be assumed that these changes indicate fundamental alterations in the fatty metabolism in the RCC carcinogenesis that warrants further study with regard to a potential target in RCC.

## Competing interests

The authors declare that they have no competing interests.

## Authors' contributions

AT coordinated the study, performed western blots and statistical analyses and wrote the paper. MoJ performed the RT-PCR and contributed to the study design. ML, MaJ and KM provided samples and clinico-pathological data. KJ supported statistical analyses and revised the paper. GK and HM performed immunohistological analyses, wrote and revised the paper. All authors read and approved the final manuscript.

## Pre-publication history

The pre-publication history for this paper can be accessed here:

http://www.biomedcentral.com/1471-2407/9/248/prepub
